# Risk of new-onset diabetes across individual statins in secondary prevention: results from the Korean national health insurance service cohort

**DOI:** 10.3389/fcvm.2026.1722107

**Published:** 2026-03-10

**Authors:** Eun Jin Park, Yoonjee Park, Dong Oh Kang, Soohyung Park, Seung-Young Roh, Jin Oh Na, Jin Won Kim, Eung Ju Kim, Seung-Woon Rha, Chang Gyu Park, Cheol Ung Choi

**Affiliations:** Cardiovascular Center, Division of Cardiology, Department of Internal Medicine, Korea University Guro Hospital, Korea University College of Medicine, Seoul, Republic of Korea

**Keywords:** atherosclerotic cardio-cerebral vascular diseases (ASCVD), National Health Insurance Service (NHIS), new onset diabetes mellitus, secondary prevention, statin, statin intensity

## Abstract

**Introduction:**

Statins are the cornerstone of secondary prevention in atherosclerotic cardiovascular disease (ASCVD), but their association with new-onset diabetes mellitus (NODM) remains incompletely defined. Whether the risk of NODM differs among individual statins within the same intensity class has not been well established.

**Methods:**

Using the Korean National Health Insurance Service (NHIS) database, we identified 29,826 patients with established ASCVD who initiated statin therapy between 2009 and 2012 and were followed for up to five years. The primary endpoint was incident NODM, defined by new diagnostic coding plus antidiabetic medication use after a three-year window period. The secondary endpoint was major adverse cardiac and cerebrovascular events (MACCE).

**Results:**

Overall, 11,918 patients (40.0%) developed NODM. The incidence of NODM was comparable between high- and moderate-intensity statins (42.9% vs. 41.1%). In the moderate-intensity group, rosuvastatin (adjusted HR 1.07, 95% CI 1.01–1.14), pravastatin (HR 1.19, 95% CI 1.02–1.38), and simvastatin (HR 1.15, 95% CI 1.06–1.20) were associated with higher NODM risk compared with atorvastatin, while fluvastatin and pitavastatin showed no significant differences. MACCE incidence was similar across statins.

**Discussion:**

In this nationwide secondary prevention cohort, the risk of NODM differed across individual statins despite comparable cardiovascular outcomes. These findings suggest that the diabetogenic effect of statins may be agent-specific rather than a uniform class effect, highlighting the importance of individualized statin selection balancing metabolic and cardiovascular benefits.

## Introduction

Statins remain the cornerstone of atherosclerotic cardiovascular disease (ASCVD) prevention (1, 2), with extensive evidence from randomized controlled trials demonstrating substantial reductions in cardiovascular events (3–5) and even regression of atherosclerotic plaque burden (6–8). However, a consistent body of evidence also indicates that statins may increase the risk of new-onset diabetes mellitus (NODM), particularly with higher-intensity regimens. Meta-analyses and large-scale trials have reported a dose-dependent relationship between statin therapy and incident diabetes, raising ongoing debate about the balance between cardiovascular benefits with metabolic risks (9, 10).

Although the overall cardioprotective effects of statins clearly outweigh the diabetogenic risk, several important questions remain unresolved. First, while high-intensity statins are known to increase the risk of NODM compared with low-intensity statins or placebo, it is unclear whether individual statins within the same intensity category confer differential metabolic risks. Second, the risk of NODM is not uniform across all patients and may be influenced by baseline metabolic risk, statin dose, and treatment duration—factors that are particularly relevant in Asian populations, who differ from Western cohorts in body composition and insulin resistance profiles. Third, data on statin-associated NODM in patients with cerebrovascular disease are limited, despite the fact that stroke survivors constitute a major proportion receiving long-term statin therapy.

To address these gaps, we conducted a nationwide, population-based analysis using the Korean National Health Insurance Service (NHIS) database. This study aimed to evaluate the incidence of NODM according to statin intensity and compare the risk of NODM among individual statins within the same intensity category, in a high-risk cohort comprising patients with coronary or cerebrovascular disease.

## Materials and methods

### Data source

This study utilized data from the Korean National Health Insurance Service (NHIS) database, which provides comprehensive health information for more than 98% of the Korean population under a mandatory universal health insurance system. The NHIS database comprises two major components. The first is the claims database, which contains patient demographics, diagnostic codes based on the International Classification of Diseases, 10th Revision (ICD-10), inpatient and outpatient visits records, prescription data, and procedural records. The second is the national health screening database, which is offered biennially to all insured individuals aged ≥40 years and includes standardized anthropometric and laboratory measurements — such as body mass index (BMI), blood pressure, fasting glucose, lipid profiles, along with self-reported lifestyle factors— such as smoking status, alcohol consumption, and physical activity. Each beneficiary is assigned a unique resident registration number, allowing longitudinal linkage between claims and screening data across healthcare institutions. The study protocol was reviewed and approved for exemption by the Institutional Review Board of Korea University Guro Hospital.

### Study populations

We identified all patients newly prescribed statins between January 2009 and December 2012 in the NHIS database. The date of the first statin prescription during this period was defined as the index date. To construct a cohort of patients receiving statins for secondary prevention, we included those with evidence of established atherosclerotic cardiovascular disease (ASCVD), defined as having ≥1 inpatient or ≥2 outpatient claims with a primary diagnosis of acute myocardial infarction, unstable angina, or stroke within one year before or at the index date (details about patient selection and ICD-10 codes are provided in Supplementary Tables S1, S2). Follow-up for all eligible patients began at the index date.

Patients were excluded if they met any of the following criteria: (1) pre-existing diabetes mellitus within one year prior to the index date; (2–4) treatment stability criteria assessed during the first year after statin initiation, including discontinuation of statin therapy for >3 consecutive months during the first year after initiation; switching to another statin type or changing statin intensity within the first year; absence of healthcare utilization for >1 year during the follow-up; (5) comorbid conditions likely to confound the diagnosis of diabetes (e.g., chronic pancreatitis, pancreatic cancer, Cushing's syndrome); (6) malignancy at baseline; (7) missing baseline covariate data from the health screening program; finally (8) use of low-intensity statin therapy at the index date.

**Figure 1:**
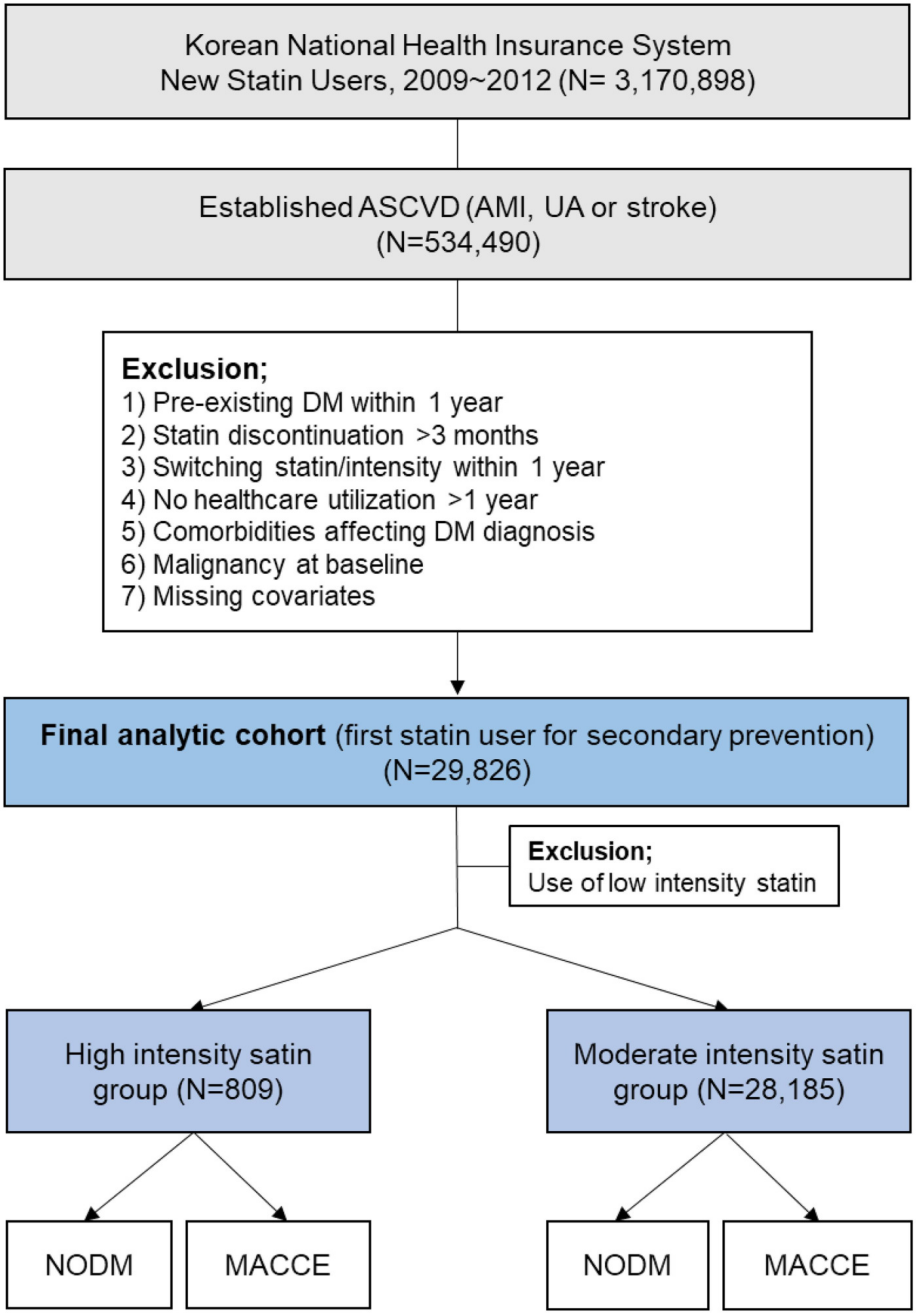
Flow diagram of study population selection. ASCVD, atherosclerotic cardiovascular disease; AMI, acute myocardial infarction; UA, unstable angina; DM, diabetes mellitus; NODM, new onset-diabetes mellitus; MACCE, major adverse cardiac and cerebrovascular events.

After applying these criteria, a total of 28,994 patients with acute myocardial infarction, unstable angina, or stroke who initiated statin therapy were included in the final analytic cohort (Figure 1). All patients were followed from the index date until the first occurrence of NODM, death, or completion of the 5-year follow-up period.

### Statin classification

All statin prescriptions recorded in the NHIS database during the study period were identified. Each statin was categorized into high- or moderate-intensity according to the 2013 ACC/AHA cholesterol treatment guidelines (Supplementary Table S3). Patients prescribed low-intensity statins were excluded from the analysis. Within each intensity category, individual statins were further analyzed separately to evaluate potential differences in the risk of NODM.

### Study outcomes

Patients were followed for up to five years from the index date of statin initiation until the earliest occurrence of and outcome event, death, or December 31, 2018. Participants were censored at the time of NODM diagnosis, death, or the end of the study period, whichever came first.

The primary endpoint was the development of NODM. To reduce misclassification and account for potential diagnostic delay, the first three years after statin initiation were designated as a window period. NODM was defined as the coexistence of a new diagnostic code for diabetes and a prescription for at least one antidiabetic medication during the follow-up. The secondary endpoint was the occurrence of major adverse cardiac and cerebrovascular events (MACCE), defined as a composite of myocardial infarction, ischemic stroke, intracranial hemorrhage, and all-cause mortality. Outcomes were identified using validated definitions from prior NHIS-based studies. Inclusion of MACCE as a secondary endpoint provided clinical context for metabolic risk of statins relative to their established cardiovascular benefits.

### Statistical analysis

Categorical variables were expressed as frequencies and percentages, and continuous variables as means with standard deviations (SD). Crude incidence rates were calculated as the number of events per 100 person-years, with 95% confidence intervals (CIs) based on a Poisson distribution. Cox proportional hazards regression models were used to estimate hazard ratios (HRs) and 95% CIs for the risk of NODM. Multivariable models were adjusted for potential confounders, including age, sex, year of cohort entry, body mass index (BMI), systolic blood pressure, fasting glucose, alanine aminotransferase (ALT), low density lipoprotein (LDL) cholesterol level, history of heart failure, hypertension, and dyslipidemia, Charlson comorbidity index, and history of alcohol and tobacco use. The proportional hazards assumption was evaluated using Schoenfeld residuals. Cumulative incidence curves were generated using the Kaplan–Meier methods, and differences between groups were assessed with log-rank test. Statistical significance was defined as a two-sided *p*-value < 0.05. All analyses were conducted using SAS software, version 9.4 (SAS Institute, Cary, NC, USA).

## Results

### Baseline characteristics

A total of 29,826 patients were included as first-line statin users for secondary prevention, of whom 809 (2.7%) received high-intensity statins and 29,017 (97.3%) received moderate-intensity statins, while 832 patients prescribed low-intensity statins were excluded from the analysis (Figure 1). The mean age of study population was 60 ± 11 years, and 49% were men. The mean follow-up duration was 2.3 ± 1.3 years (median 2.1 years) for the primary outcome analysis and 3.3 ± 1.8 years (median 3.1 years) for secondary outcome. Baseline characteristics were generally comparable between the high- and moderate-intensity groups. BMI, systolic blood pressure, fasting glucose, and lipid parameters showed no statistically significant differences according to statin intensity (Table 1). The mean LDL-C and total cholesterol levels were 132 mg/dL and 198 mg/dL, respectively, at baseline, reflecting suboptimal lipid control before statin initiation. The mean fasting glucose level was 97 mg/dL, and the average BMI was 24 kg/m^2^, consistent with a typical east asian secondary prevention population. Regarding comorbidities, approximately 44% of patients had hypertension, 42% had dyslipidemia, and 4.4% had a history of heart failure. The Charlson comorbidity index distribution did not differ significantly between groups. Smoking and alcohol consumption patterns were also similar, with 37% of patients being current or former smokers and nearly half reporting light alcohol use (<2 times per week). Among individual statins, atorvastatin was the most frequently prescribed agent (*n* = 19,661, 66%), followed by simvastatin (*n* = 4,565, 15%) and rosuvastatin (*n* = 3,062, 10%). Detailed baseline characteristics according to individual statins within each intensity group are provided in Supplementary Table S4.

**Table 1:** Baseline characteristics of each intensity group.

(*N*, %) or (mean, SD)	High-intensity group	Moderate-intensity group	*P*-value
*N*	(*n* = 809)	(*n* = 28,185)	(*n* = 28,994)
Age	60.40 (±11.90)	60.62 (±11.43)	0.59
BMI, kg/m^2^	24.87 (±2.90)	24.63 (±3.12)	0.04
SBP, mmHg	128.29 (±16.09)	127.38 (±15.97)	0.11
Fasting glucose, mg/dL	97.43 (±15.83)	97.42 (±16.93)	0.98
Total cholesterol, mg/dL	212.00 (±47.82)	215.83 (±48.39)	0.10
ALT, U/L	28.80 (±63.07)	26.76 (±23.83)	0.03
LDL-C, mg/dL	133.32 (±100.80)	132.17 (±63.67)	0.62
Male sex	520 (64.28%)	137,44 (48.76%)	<0.001
Charlson comorbidity index			
0	293 (36.22%)	9,054 (32.12%)	0.08
1	513 (63.41%)	18,968 (67.30%)	0.08
2	2 (0.25%)	124 (0.44%)	0.08
≥3	1 (0.12%)	39 (0.14%)	0.08
Alcohol			
0–1 per week	588 (72.68%)	21,913 (77.75%)	0.003
2–3 per week	143 (17.68%)	4,180 (14.83%)	0.003
≥4 per week	78 (9.64%)	2,092 (7.42%)	0.003
Smoking			
Never smoker	433 (53.52%)	17,811 (63.19%)	<0.001
Ex- or current smoker	376 (46.48%)	10,374 (36.81%)	<0.001
Heart failure	44 (5.44%)	1,226 (4.35%)	0.14
Hypertension	415 (51.30%)	12,400 (44.00%)	<0.001
Dyslipidemia	399 (49.32%)	12,039 (42.71%)	<0.001

BMI, body mass index; SBP, systolic blood pressure; ALT, alanine transaminase; LDL-c, low density lipoprotein cholesterol; n, number; SD, standard deviation.

### Primary outcome: new-onset diabetes mellitus (NODM)

During follow-up, 11,918 patients (40.0%) developed NODM. To minimize the bias related to statin intensity and type, comparisons were made between individual statins within the same intensity group, under the assumption that agents of the same intensity have comparable lipid-lowering potency and that patients taking the same intensity share similar baselin risk profiles. The overall cumulative incidence of NODM was comparable between high- and moderate-intensity groups (42.9% vs. 41.1%, respectively; Table 2).

**Table 2:** Primary outcome: NODM according to statin type.

High intensity	*N* (%)	NODM	Risk (%)	Total PY	Incidence rate/100PY	Crude HR (95% CI)	*P*-value	Adjusted HR (95%CI)	*P*-value
Total	809	347	42.9						
Atorvastatin	485 (60)	199	41.0	1,981.48	100.43	1		1	
Rosuvastatin	324 (40)	148	45.7	1,264.21	117.07	1.17 (0.94–1.44)	0.16	1.16 (0.93–1.44)	0.18
Moderate intensity	*N* (%)	NODM	Risk (%)	Total PY	Incidence rate/100PY	Crude HR (95% CI)	*P*-value	Adjusted HR (95%CI)	*P*-value
Total	28,185	11,571	41.1						
Atorvastatin	19,176 (68.0%)	7,727	40.3	80,541.16	95.94	1		1	
Rosuvastatin	2,738 (9.7%)	1,132	41.3	11,184.62	101.21	1.06 (0.99–1.13)	0.08	1.07 (1.01–1.14)	0.03
Fluvastatin	259 (0.9%)	106	40.9	1,042.75	101.65	1.06 (0.88–1.29)	0.52	1.10 (0.91–1.33)	0.35
Pitavastatin	1,218 (4.3%)	509	41.8	4,954.45	102.74	1.07 (0.98–1.17)	0.12	1.07 (0.98–1.17)	0.14
Pravastatin	408 (1.4%)	177	43.4	1,612.48	109.77	1.15 (0.99–1.34)	0.06	1.19 (1.02–1.38)	0.03
Simvastatin	4,386 (15.6%)	1,920	43.8	17,628.89	108.91	1.14 (1.08–1.19)	<0.001	1.15 (1.06–1. 20)	<0.001

NODM, new onset-diabetes mellitus; n, number; PY, person-year; HR, hazard ratio; CI, confidence interval.

In the high-intensity group (*n* = 809), 347 patients developed NODM. Compared with atorvastatin, rosuvastatin showed numerically higher incidence rate (117.1 vs. 100.4 per 100 person-years), though this difference did not reach statistical significance (adjusted HR 1.16, 95% CI 0.93–1.44, *p* = 0.18).

In the moderate-intensity group (*n* = 28,185), 11,571 patients developed NODM. Atorvastatin served as the reference. Rosuvastatin was associated with a significantly higher risk (absolute risk 41.3%, adjusted HR 1.07, 95% CI 1.01–1.14; *p* = 0.03), as were pravastatin (absolute risk 43.4%, HR 1.19, 95% CI 1.02–1.38; *p* = 0.03) and simvastatin (absolute risk 43.8%, HR 1.15, 95% CI 1.06–1.20; *p* < 0.001). In contrast, fluvastatin and pitavastatin showed no significant differences relative to atorvastatin (adjusted HR 1.10, 95% CI 0.91–1.33, *p* = 0.35 and 1.07, 95% CI 0.98–1.17; *p* = 0.14, respectively). Kaplan–Meier curves for the cumulative incidence of NODM or moderate-intensity group are presented in Supplementary Figure S1.

Subgroup analyses were performed according to predefined variables, including age, sex, baseline LDL-C, smoking status, alcohol intake, and BMI (Supplementary Figure S2). The association between moderate-intensity statins and NODM risk appeared generally similar across subgroups, and no statistically significant interaction effects were detected. In most strata, rosuvastatin, pravastatin, and simvastatin tended to show higher risks of NODM compared with atorvastatin. The magnitude of excess risk appeared greater among patients with higher baseline LDL-C levels, current smokers, and those with BMI ≥ 25 kg/m^2^. However, given the relatively small sample sizes in certain subgroups, these analyses may have been underpowered to detect modest interaction effects. Therefore, subgroup findings should be considered exploratory and interpreted with caution. Overall, the results suggest that the observed associations were broadly consistent across demographic and metabolic backgrounds.

### Secondary outcome: cardiocerebrovascular events

During follow-up, a total of 6,366 patients experienced MACCE. Event were compared according to statin intensity (Table 3).

**Table 3:** Secondary outcome: MACCE according to statin type.

High intensity	*N* (%)	MAC(C)E	Risk (%)	Total PY	Crude HR (95% CI)	*P*-value	Adjusted HR (95%CI)	*P*-value
Total	809	226	27.9					
Atorvastatin	485 (40)	149	30.7	2,202.71	1		1	
Rosuvastatin	324 (60)	77	23.8	1,537.48	0.74 (0.56–0.98)	0.03	0.75 (0.57–1.00)	0.046
Moderate intensity	*N* (%)	MAC(C)E	Risk (%)	Total PY	Crude HR (95% CI)	*P*-value	Adjusted HR (95%CI)	*P*-value
Total	28,185	6,140						
Atorvastatin	19,176 (68.0%)	4,169	21.7	94,698.88	1		1	
Rosuvastatin	2,738 (9.7%)	587	21.4	13,455.52	0.99 (0.91–1.08)	0.83	0.99 (0.91–1.08)	0.80
Fluvastatin	259 (0.9%)	58	22.4	1,261.31	1.05 (0.81–1.36)	0.73	1.04 (0.80–1.34)	0.79
Pitavastatin	1,218 (4.3%)	258	21.2	6,084.74	0.96 (0.84–1.08)	0.48	0.96 (0.8–1.09)	0.57
Pravastatin	408 (1.4%)	78	19.1	2,028.38	0.87 (0.70–1.09)	0.24	0.9 (0.72–1.13)	0.36
Simvastatin	4,386 (15.6%)	990	22.6	21,489.08	1.05 (0.98–1.12)	0.18	1.06 (0.99–1.13)	0.11

MAC(C)E, major adverse cardiac and cerebrovascular events; n, number; PY, person-year; HR, hazard ratio; CI, confidence interval.

In the high-intensity group, rosuvastatin was associated with a numerically lowe incidence of MACCE than atorvastatin (77 vs. 149 events), with borderline statistical significance (adjusted HR 0.75, 95% CI 0.57–1.00; *p* = 0.046).

In the moderate-intensity group (*n* = 28,185), 6,140 patients experienced MACCE. When compared with atorvastatin, rosuvastatin, fluvastatin, pitavastatin, and pravastatin demonstrated comparable risks of MACCE (all *p* > 0.05), whereas simvastatin showed a modest but nonsignificant trend toward higher risk (adjusted HR 1.06, 95% CI 0.99–1.13; *p* = 0.11).

Overall, no meaningful difference in cardiovascular and cerebrovascular outcomes was observed among individual statins within the same intensity category, suggesting that once LDL-C reduction is achieved, the choice of statin type may exert limited influence on long-term atherosclerotic outcomes.

### Net clinical implications: integration of metabolic and cardiovascular outcomes

**Figure 2:**
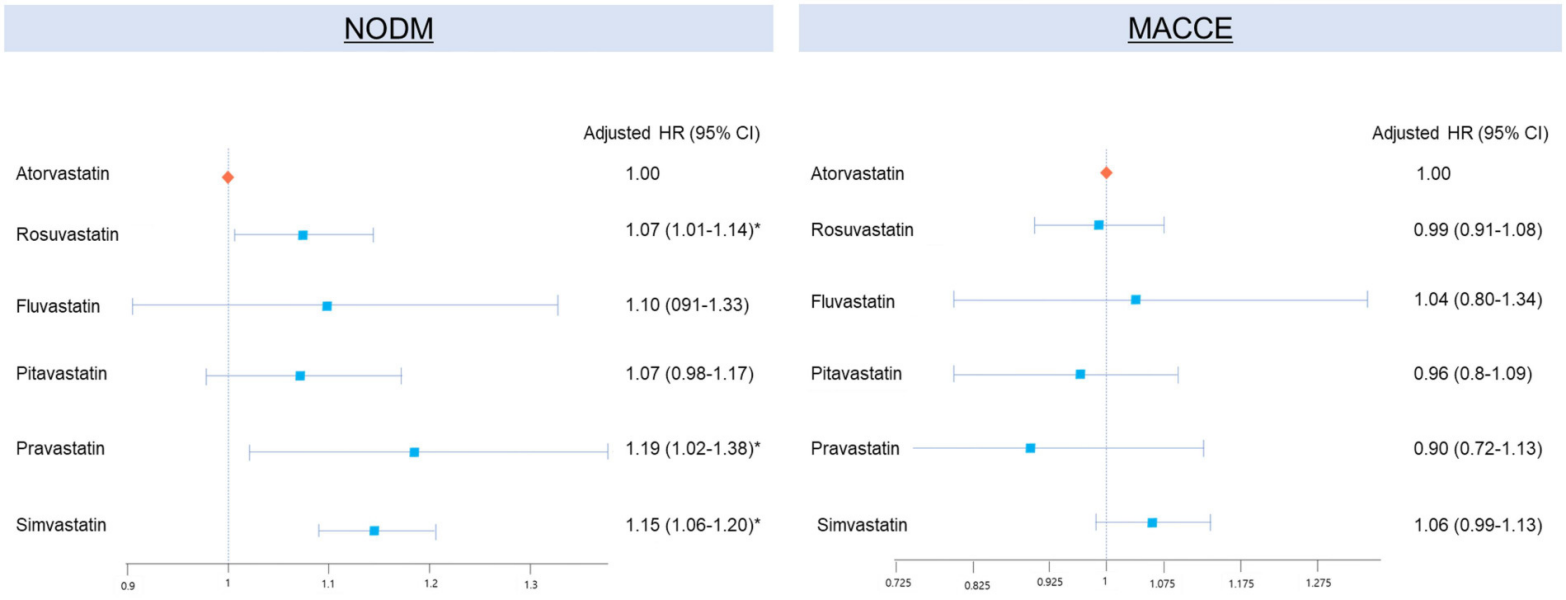
Integrated forest plot of statin-specific risks for NODM and MACCE; Among moderate-intensity statins. CVD, cardiovascular disease; NODM, new-onset diabetes mellitus; MACCE, major adverse cardiac and cerebrovascular events; HR, hazard ratio; CI, confidence interval. (*indicates clinical significance.).

To integrate both metabolic and cardiovascular perspectives, a combined forest plot was constructed comparing adjusted hazard ratios for NODM and MACCE across individual moderate-intensity statins (Figure 2). As illustrated, rosuvastatin, pravastatin, and simvastatin were associated with a relatively higher risk of NODM compared with atorvastatin, whereas cardiovascular outcomes were broadly similar. This pattern suggests a divergence between metabolic and vascular effects rather than a consistent adverse pattern. Notably, although rosuvastatin showed a modest increase in NODM incidence, its cardiovascular protection remained noninferior, indicating that the net clinical benefit of statin therapy was largely preserved within the moderate-intensity class.

## Discussion

In this large, nationwide cohort of Korean patients treated with statins for secondary prevention, we found that the risk of NODM differed significantly among individual statins, even within the same intensity class. While the overall incidence of NODM was comparable between high- and moderate-intensity users, rosuvastatin, pravastatin, and simvastatin were associated with a higher risk of NODM than atorvastatin within the moderate-intensity group. In contrast, cardiovascular outcomes, including MACCE, were broadly similar across statins, suggesting preserved cardioprotective efficacy despite heterogeneous metabolic effects.

### Comparison with landmark RCTs and recent individual patient-data meta-analyses

The association between statin therapy and incident diabetes has been consistently demonstrated in randomized trials and meta-analyses. *post hoc* analyses of major cardiovascular outcome trials, including JUPITER (11), TNT (12), IDEAL (13), and SPARCL (14), as well as the recent individual participant data meta-analysis from the CTT Collaboration (15), reported a dose- or intensity-dependent increase in diabetes risk. These findings have generally been interpreted as a class-wide effect of statins. However, most prior studies pooled different statins together or compared treatment intensity rather than directly evaluating individual agents, limiting their ability to detect potential heterogeneity between specific drugs.

### Heterogeneity across individual statins within the same intensity

In contrast, our findings suggest that the metabolic effects of statins may not be entirely uniform within the same therapeutic intensity. Although previous studies have largely emphasized a class- or intensity-dependent increase in diabetes risk, our results indicate that the magnitude of diabetogenic risk may differ across individual agents even when comparable lipid-lowering intensity is used. Importantly, cardiovascular protection remained similar across statins, suggesting that differences, if present, may relate more to metabolic safety profiles rather than efficacy.

The mechanisms underlying these differences are likely multifactorial and remain incompletely understood. Statins have been reported to influence insulin sensitivity by downregulating GLUT-4 expression in skeletal muscle and pancreatic β-cell function through shared pathways (16, 17), which may explain the modest class-wide increase in diabetes risk observed in prior studies. At the same time, pharmacologic characteristics—such as lipophilicity, potency, tissue distribution, and hepatic selectivity—vary among individual statins and could plausibly contribute to differential metabolic effects (18). Therefore, rather than reflecting a purely uniform class effect, statin-associated dysglycemia may represent heterogeneous responses across agents within the same intensity category.

### Unique contributions and clinical implications

This study provides several unique contributions. First, by leveraging a nationwide real-world database, we were able to directly compare individual statins within the same intensity class, offering head-to-head evidence that is rarely available from randomized trials. Second, our findings suggest that while cardiovascular protection appears consistent across agents, metabolic safety may differ, which has practical implications for personalized lipid management. Importantly, our cohort consisted exclusively of patients with established ASCVD receiving statins for secondary prevention, and all participants were treated with at least moderate-intensity therapy. Therefore, our findings are most applicable to high- or very-high-risk patients in whom moderate- to high-intensity statins are routinely recommended. In patients at high metabolic risk—such as those with obesity, impaired fasting glucose, or metabolic syndrome—clinicians may consider closer glycemic monitoring and individualized statin selection when initiating or intensifying therapy. These results support a more tailored approach that balances cardiovascular benefit with metabolic safety, particularly in high- or very-high-risk populations.

## Limitations

Several limitations should be acknowledged. First, detailed information on concomitant use of non-statin lipid-lowering agents, such as ezetimibe or other combination therapies, was not available in this claims database. However, during the study period (2009–2012), these agents were not routinely recommended as first-line or combination therapy in Korea and were likely prescribed infrequently; thus, their impact on our findings is expected to be limited. Second, important lifestyle and genetic factors—including diet, physical activity, and family history—were not captured and may have confounded both statin selection and diabetes risk. Third, although the overall cohort size was large, the number of patients receiving high-intensity statins and certain individual statins was relatively small, which may have limited statistical power and increased the possibility of type II error in subgroup analyses; therefore, these findings should be interpreted cautiously. This distribution also reflects real-world prescribing patterns in East Asian practice, where high-intensity statins remain underutilized despite contemporary international guidelines (1, 2, 19, 20) and major outcome trials (21, 22) supporting early use of maximally tolerated high-intensity therapy for secondary prevention in patients with established ASCVD. Similarly, the small sample sizes of several moderate-intensity statin subgroups likely mirror their lower prescription rates in routine clinical practice, which should be considered when interpreting subgroup results. Fourth, although baseline glucose levels were available, prediabetes or impaired fasting glucose was not specifically defined or adjusted for in the analysis. Prior studies, including *post hoc* analyses of JUPITER, have shown that incident diabetes occurs more frequently among individuals with pre-existing metabolic susceptibility. Therefore, differences in baseline glycemic risk across statin groups may not have been fully captured, and residual confounding related to subclinical dysglycemia cannot be excluded. Finally, because this study was conducted using a nationwide Korean population, generalizability to non-Asian populations should be interpreted with caution.

## Data Availability

The data analyzed in this study is subject to the following licenses/restrictions: the NHIS data are restricted under Korean privacy law and cannot be publicly shared. Access requires prior approval from the NHIS. Requests to access these datasets should be directed to NHIS data portal at https://nhiss.nhis.or.kr or via email to nhiss@nhis.or.kr, and will be granted only through legitimate procedures and upon official approval by the NHIS.
