# Supplementary material for: Risk of new-onset diabetes across individual statins in secondary prevention: results from the Korean national health insurance service cohort
Source: Front Cardiovasc Med. 2026 Mar 10;13:1722107. doi: 10.3389/fcvm.2026.1722107 (PMC13008627; doi:10.3389/fcvm.2026.1722107)
Supplement: Supplementary file 1 [file Datasheet1.docx]

**Supplementary Data**

**Supplementary Table 1. Patient selection chart**

|  |  | **Exclude** | **N** |
| --- | --- | --- | --- |
| **Population** | First statin prescription during 2009-2012 |  | 3,170,898 |
|  | Exclude 1yr of wachout | 35,190 | 3,135,708 |
| **Inclusion** | AMI |  |  |
|  | Unstable angina |  |  |
|  | Ischemic stroke |  |  |
|  | Hemorrahagic stroke |  | 534,490 |
| **Exclusion** | Previous DM | 262,593 | 271,897 |
|  | Discomtinuation of statin for >3 consecutive months |  | 79,671 |
|  | Malignancy |  | 69,639 |
|  | No healthcare use for >1yr |  | 63,489 |
|  | Crossover in statin type within 1yr |  | 40,865 |
|  | Missing confounder |  | **29,826** |
|  | Use of low intentity statin |  | **28,994** |
|  |  |  |  |

**Supplmentary Table 2. Difinition and Diagnostic Code**

| Diagnosis | Definition | Code |
| --- | --- | --- |
| Diabetes mellitus | defined as at least one claim with a diabetes diagnostic code **or**  a prescription for antidiabetic medication within one year prior to the index date | E10, E11, E12, E13, E14 |
| Prior myocardial infarction | defined as ≥1 inpatient or ≥2 outpatient claims **and**  diagnostic codes | I21, I22, I25.2 |
| Unstable angina | defined as ≥1 inpatient or ≥2 outpatient claims **and**  diagnostic codes | I20.0 |
| Prior ischemic stroke | defined as ≥1 inpatient or ≥2 outpatient claims **and**  diagnostic codes | I63, I64 |
| Prior intracranial hemorrhage | defined as ≥1 inpatient or ≥2 outpatient claims **and**  diagnostic codes | I60, I61, I62 |

**Supplmentary Table 3. Statin Classification**

| high-intensity | Moderate-intensity | Low-intensity |
| --- | --- | --- |
| Atorvastatin 40-80 mg | Atorvastatin 10-20 mg | Simvastatin 10 mg |
| Rosuvastatin 20-40 mg | Rosuvastatin 5-10 mg | Pravastatin 10-20 mg |
|  | Simvastatin 20-40 mg | Lovastatin 20 mg |
|  | Pravastatin 40-80 mg | Fluvastatin 20-40 mg |
|  | Lovastatin 40mg | Pitavastatin 1 mg |
|  | Fluvastatin XL 80 |  |
|  | Fluvastatin 40mg bid |  |
|  | Pitavastatin 2-4 mg |  |

**Supplmentary Table 4. Baseline characteristics as individual statin**

| (n, %) or (mean, SD) | Total Group | Rosuvastatin | Atorvastatin | Pravastatin | Simvastatin | Pitavastatin | Fluvastatin | P-value |
| --- | --- | --- | --- | --- | --- | --- | --- | --- |
| n |  | (n = 3,062) | (n = 19,661) | (n = 408) | (n = 4,386) | (n = 1,218) | (n = 259) | (n = 29,826) |
| Age (y) | 60.61 ± 11.44 | 60.76 ± 11.43 | 60.69 ± 11.43 | 60.21 ± 11.15 | 60.11 ± 11.65 | 60.92 ± 10.93 | 61.04 ± 11.59 | 0.04 |
| BMI, kg/m^2^ | 24.64 ± 3.12 | 24.61 ± 3.15 | 24.65 ± 3.12 | 24.73 ± 3.17 | 24.62 ± 3.10 | 24.52 ± 3.09 | 24.68 ± 3.03 | 0.72 |
| SBP, mmHg | 127.40 ± 15.98 | 127.35 ± 15.96 | 127.44 ± 15.92 | 126.77 ± 16.78 | 127.40 ± 16.04 | 127.26 ± 15.95 | 126.56 ± 18.02 | 0.90 |
| Fasting glucose, mg/dL | 97.42 ± 16.90 | 97.36 ± 16.50 | 97.25 ± 16.62 | 97.05 ± 13.87 | 98.29 ± 18.21 | 97.17 ± 18.55 | 97.61 ± 15.14 | 0.02 |
|  |  |  |  |  |  |  |  |  |
| Total cholesterol, mg/dL | 215.75 ± 48.38 | 204.43 ± 45.21 | 217.30 ± 45.82 | 203.14 ± 42.51 | 220.45 ± 61.68 | 207.22 ± 38.13 | 212.38 ± 41.82 | < 0.001 |
| ALT, U/L | 26.82 ± 25.75 | 27.02 ± 35.89 | 26.86 ± 25.55 | 27.10 ± 23.30 | 26.73 ± 19.42 | 26.30 ± 20.55 | 25.55 ± 15.75 | 0.92 |
| LDL-C, mg/dL | 132.21 ± 65.00 | 126.73 ± 87.85 | 133.13 ± 59.61 | 120.91 ± 35.13 | 134.73 ± 73.15 | 125.83 ± 57.92 | 132.20 ± 52.74 | < 0.001 |
| Male sex | 14,264 (49.20%) | 1,770 (57.81%) | 9,449 (48.06%) | 217 (53.19%) | 2082 (47.47%) | 622 (51.07%) | 124 (47.88%) | < 0.001 |
| Charlson comorbidity index |  |  |  |  |  |  |  |  |
| 0 | 9,347 (32.24%) | 1,125 (36.74%) | 6,328 (32.19%) | 139 (34.07%) | 1,275 (29.07%) | 388 (31.86%) | 92 (35.52%) | < 0.001 |
| 1 | 19,481 (67.19%) | 1,914 (62.51%) | 13,228 (67.28%) | 267 (65.44%) | 3,079 (70.20%) | 826 (67.82%) | 167 (64.48%) | < 0.001 |
| 2 | 126 (0.44%) | 19 (0.62%) | 76 (0.38%) | 2 (0.49%) | 25 (0.57%) | 4 (0.33%) | 0 (0%) | < 0.001 |
| ≥ 3 | 40 (0.14%) | 4 (0.13%) | 29 (0.15%) | 0 (0%) | 7 (0.16%) | 0 (0%) | 0 (0%) | < 0.001 |
| Alcohol |  |  |  |  |  |  |  |  |
| 0–1 per week | 22,501 (77.61%) | 2,359 (77.04%) | 15,289 (77.76%) | 327 (80.15%) | 3,376 (76.97%) | 943 (77.42%) | 207 (79.92%) | 0.54 |
| 2–3 per week | 4,323 (14.91%) | 487 (15.90%) | 2,912 (14.81%) | 55 (13.48%) | 652 (14.87%) | 185 (15.19%) | 32 (12.36%) | 0.54 |
| ≥ 4 per week | 2,170 (7.48%) | 216 (7.05%) | 1,460 (7.43%) | 26 (6.373%) | 358 (8.16%) | 90 (7.39%) | 20 (7.72%) | 0.54 |
| Smoking |  |  |  |  |  |  |  |  |
| Never smoker | 18,244 (62.92%) | 1,759 (57.45%) | 12,545 (63.81%) | 250 (61.28%) | 2,772 (63.20%) | 742 (60.92%) | 176 (67.95%) | < 0.001 |
| Ex- or current smoker | 10,750 (37.08%) | 1,303 (42.55%) | 7,116 (36.19%) | 158 (38.73%) | 1,614 (36.80%) | 476 (39.08%) | 83 (32.05%) | < 0.001 |
| Heart failure | 1,270 (4.38%) | 187 (6.11%) | 824 (4.19%) | 23 (5.64%) | 153 (3.49%) | 64 (5.26%) | 19 (7.34%) | < 0.001 |
| Hypertension | 12,815 (44.20%) | 1,630 (53.23%) | 8,576 (43.62%) | 199 (48.78%) | 1,665 (37.96%) | 629 (51.64%) | 116 (44.79%) | < 0.001 |
| Dyslipidemia | 12,438 (42.90%) | 1,496 (48.86%) | 8,377 (42.61%) | 178 (43.63%) | 1,744 (39.76%) | 555 (45.57%) | 88 (33.98%) | < 0.001 |
|  |  |  |  |  |  |  |  |  |

BMI: Body mass index, SBP: Systolic blood pressure, ALT: Alanine transaminase, LDL-C: Low-density lipoprotein cholesterol, n: number, SD: standard deviation

**Supplmentary Figure 1. Kaplan-Meier curves for the cumulative incidence of NODM**

**
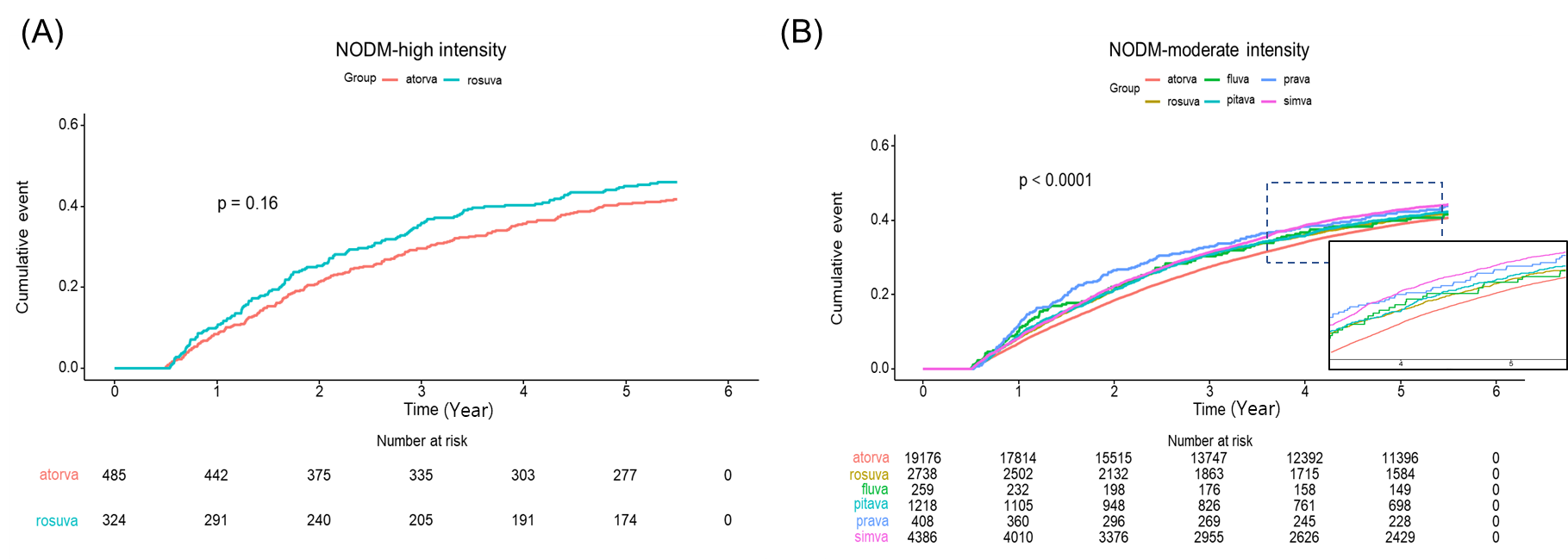
**

NODM; New-onset diabetes mellitus, atorva; atorvastatin, rosuva; rosuvastatin, fluva; fluvastatin, pitava: pitavastatin, prava: pravastatin, simva: simvastatin.

**Supplementary Figure 2. Subgroup Analysis of New-Onset Diabetes Mellitus (NODM) Comparing Individual Moderate-Intensity Statins**


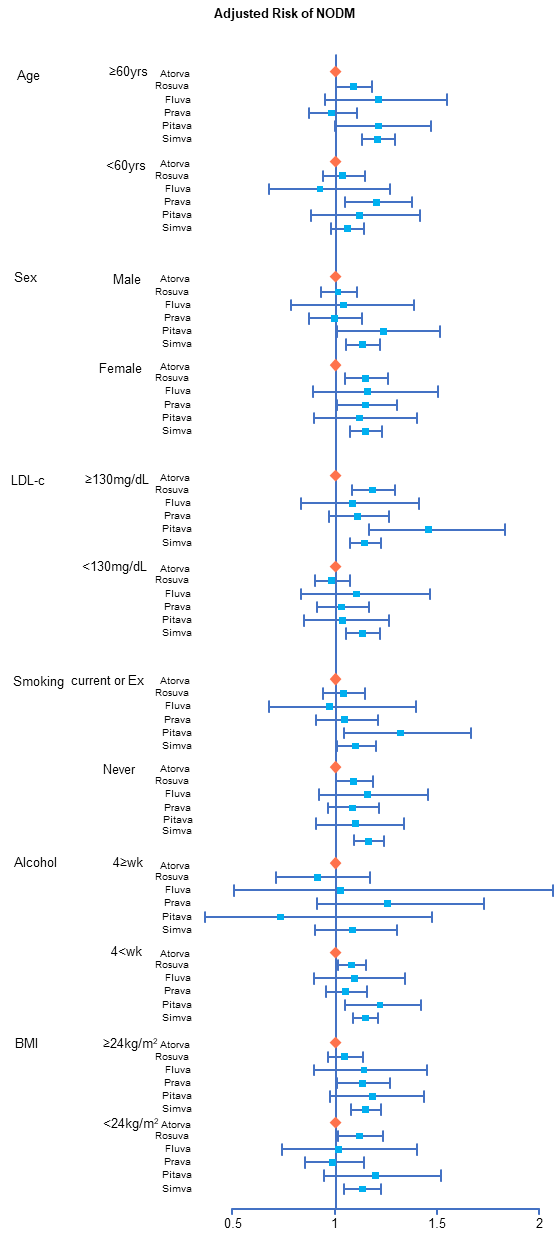


NODM; New-onset diabetes mellitus, LDL-C; Low-density lipoprotein cholesterol, BMI; Body mass index, atorva; atorvastatin, rosuva; rosuvastatin, fluva; fluvastatin, pitava: pitavastatin, prava: pravastatin, simva: simvastatin.
